# Altered regional homogeneity and functional connectivity of brain activity in young HIV-infected patients with asymptomatic neurocognitive impairment

**DOI:** 10.3389/fneur.2022.982520

**Published:** 2022-10-10

**Authors:** Shuai Han, Xire Aili, Juming Ma, Jiaojiao Liu, Wei Wang, Xue Yang, Xi Wang, Lijun Sun, Hongjun Li

**Affiliations:** ^1^Department of Radiology, Beijing Youan Hospital, Capital Medical University, Beijing, China; ^2^Beijing Advanced Innovation Centre for Biomedical Engineering, Beihang University, Beijing, China; ^3^STD & AIDS Clinic, Department of Infectious Diseases, Beijing Youan Hospital, Capital Medical University, Beijing, China

**Keywords:** HIV, asymptomatic neurocognitive impairment, resting-state functional MRI, regional homogeneity, functional connectivity

## Abstract

**Objective:**

Asymptomatic neurocognitive impairment (ANI) is a predominant form of cognitive impairment in young HIV-infected patients. However, the neurophysiological mechanisms underlying this disorder have not been clarified. We aimed to evaluate the altered patterns of functional brain activity in young HIV-infected patients with ANI by quantifying regional homogeneity (ReHo) and region of interest (ROI)-based functional connectivity (FC).

**Methods:**

The experiment involved 44 young HIV-infected patients with ANI and 47 well-matched healthy controls (HCs) undergoing resting-state functional magnetic resonance imaging (rs-fMRI) and neurocognitive tests. Reho alterations were first explored between the ANI group and HC groups. Subsequently, regions showing differences in ReHo were defined as ROIs for FC analysis. Finally, the correlation of ReHo and FC with cognitive function and clinical variables was assessed.

**Results:**

Compared with HCs, ANI patients had a significant ReHo decrease in the right lingual gyrus (LING. R), right superior occipital gyrus (SOG. R), left superior occipital gyrus (SOG. L), left middle occipital gyrus (MOG. L), right middle frontal gyrus (MFG. R), cerebellar vermis, ReHo enhancement in the left middle frontal gyrus (MFG. L), and left insula (INS L). The ANI patients showed increased FC between the LING. R and MOG. L compared to HC. For ANI patients, verbal and language scores were negatively correlated with increased mean ReHo values in the MFG.L. Increased mean ReHo values in the INS. L was positively correlated with disease duration—the mean ReHo values in the LING. R was positively correlated with the abstraction and executive function scores. Increased FC between the LING. R and MOG. L was positively correlated with verbal and language performance.

**Conclusion:**

The results suggest that the visual network might be the most vulnerable area of brain function in young HIV-infected patients with ANI. The middle frontal gyrus, cerebellar vermis, and insula also play an important role in asymptomatic neurocognitive impairment. The regional homogeneity and functional connectivity of these regions have compound alterations, which may be related to the course of the disease and neurocognitive function. These neuroimaging findings will help us understand the characteristics of brain network modifications in young HIV-infected patients with ANI.

## Introduction

The human immunodeficiency virus (HIV) can cross the brain barrier and infiltrate the nervous system soon after infection (1). It causes inflammatory changes in the central nervous system (CNS) and affects cognitive function. Cognitive impairment caused by HIV, known as HIV-associated neurocognitive disorder (HAND), can affect multiple cognitive domains of HIV-infected patients, such as memory, sensory, and fine motor function (2). HAND can be subdivided into asymptomatic neurocognitive impairment (ANI), mild neurocognitive disorder (MND), and HIV-associated dementia (HAD) based on its severity (3). Combined antiretroviral therapy (cART) makes HIV a chronic disease and reduces the incidence of severe HAND, but unfortunately, it does not reduce the overall incidence of HAND (4, 5). Young HIV-infected patients, although they have better self-immunity and treatment compliance, still suffer from ANI. ANI refers to the stage in which the patient's cognitive function is impaired, but it does not affect their daily life. This is a critical period during which HAND may be delayed or reversed (6). If patients are not diagnosed and intervention does not occur promptly, their cognitive ability will further decline as the disease progresses (7, 8).

However, it is difficult to accurately diagnose ANI at present. Diagnosis requires a series of neurocognitive scale tests, which are time-consuming, and the results are easily affected by various factors, such as the patient's education level, environment, and examiner (9, 10). In addition, these patients have no obvious clinical symptoms. In fact, cognitive impairment implies that neurons have been damaged and brain function has changed. However, the pathophysiological mechanism of ANI in young HIV patients and the altered patterns of functional brain activity are still unclear. Functional magnetic resonance imaging (fMRI) provides researchers with a unique perspective to study changes in brain function. It also makes it possible for us to search for neuroimaging diagnostic markers of ANI, improve our understanding of the mechanism of the injury, and promote early diagnosis and treatment.

Resting-state functional magnetic resonance imaging (rs-fMRI) is an imaging technique to study the intrinsic functional network of the brain, which is sensitive to injury-related brain changes (11, 12). The regional and connectivity information obtained by these rs-fMRI analysis methods may contribute to a better understanding of the pathophysiology of HAND and even provide useful information on HAND therapeutic targets. Several studies have reported changes in local brain areas, including the striate cortex, frontal lobe, occipital lobe, and temporal regions, that may be associated with cognitive functions, such as learning, memory, and executive function (13–16). These studies did not strictly define the patient's HAND stage, although it is crucial for us to understand the characteristics of the disease at different stages. Each region of the brain does not work independently, and assessing functional connectivity (FC) between multiple regions can reveal brain changes underlying functional damage in HIV patients. The FC of different brain regions in HAND patients may be increased or decreased, and opposite conclusions have been described in different studies (17, 18). We believe this may be related to a failure to clarify the HAND stage, given that the FC results differ at different HAND stages. In addition, current studies rarely select regions of interest (ROI) based on a priori assumptions. Due to a lack of targeting, the results of the whole-brain analysis do not reflect the real characteristics of the disease well. Notably, in some studies, the same neurocognitive test was not administered to the healthy control group (HC) to screen out those the researchers believed were normal but actually had cognitive impairment. This may affect the reliability of the results to a certain extent.

Among these rs-fMRI algorithms, the regional homogeneity (ReHo) algorithm describes the synchronization of a voxel and its adjacent voxel time series. It reflects spontaneous neural activity in local brain regions (19). The FC algorithm based on seed points calculates the time series correlation coefficient between different ROIs, which reflects the connection pattern between distant brain regions (20). These two methods are complementary in the study of functional changes in diseases and can provide more insights into disease pathology (21–23). However, as far as we know, few studies address the mechanism of ANI brain activity impairment in young HIV-infected individuals by combining these two approaches.

In this study, we tried to detect alterations in local intrinsic brain activity and regional connectivity features in young ANI patients. ReHo and ROI-based FC analysis were applied to the fMRI data of young HIV-infected patients with ANI and HCs. We speculate that HIV impairs local brain ReHo and alters FC between these deficit regions. These alterations may be related to the duration of HIV infection, the decline in CD4+ T-cell count, and cognitive performance, suggesting their role in ANI.

## Materials and methods

### Participants

This study was approved by the medical ethics committee at Beijing You'an Hospital, and all the participants gave written informed consent in accordance with the Declaration of Helsinki. From November 2020 to April 2022, 44 young HIV-infected patients with ANI and 47 HCs who were age-, gender- and education-matched were recruited for our study. Given that more than 95 percent of our patients were right-handed men and that gender and handedness can affect brain structure and function (24, 25), we set our inclusion criteria to exclude these factors that might have influenced our results. The inclusion criteria for all participants in our study were as follows: (1) Chinese male, (2) aged 20–40, and (3) right-hand dominant. The exclusion criteria for all participants included the following: (1) presence of brain tumors, infection, stroke, epilepsy, and other diseases of the nervous system that can cause cognitive impairment according to medical history or conventional imaging exams; (2) depression, anxiety, or any other psychiatric illness; (3) history of substance abuse, alcoholism, and drugs; and (4) MR contraindication. For the HIV patients, the diagnosis was based on immunoassay western blot or through PCR. Furthermore, only those infected by homosexual sex were recruited. The diagnostic classification for ANI according to the Frascati criteria meets the following four conditions: (1) performance in ≥ 2 cognitive domains at least one standard deviation below the mean of the demographic-adjusted normative scores; (2) no decline in daily life function; (3) impairment does not meet the criteria for delirium or dementia; (4) no evidence that ANI is caused by other reasons (3). All participants underwent clinical examinations and neurocognitive tests before the MR scanning. Clinical data are presented in Table 1.

**Table 1:** Demographic and clinical characteristics of the participants.

**Demographic**	**ANI (*n* = 44)**	**HC (*n* = 47)**	***p*-Value**
Age (years) (Mean ± SD)	30.2 ± 4.5	31.0 ± 6.0	0.735^a^
Education (years) (Mean ± SD)	15.2 ± 2.3	15.0 ± 3.0	0.750^b^
Disease course (months) (Mean ± SD)	45.0 ± 32.5		
Plasma VL (HIV RNA)	TND		
CD4^+^ (Mean ± SD)	516.1 ± 215.9		
CD4^+^/CD8^+^ratio (Mean ± SD)	0.60 ± 0.27		
Neurocognitive performance T scores (Mean ± SD)		
Speed of information processing	41.5 ± 8.7	47.0 ± 5.4	< 0.001^b^
Memory (learning and recall)	34.5 ± 7.1	46.3 ± 4.8	< 0.001^b^
Verbal and language	46.0 ± 7.9	52.3 ± 6.6	< 0.001^b^
Abstraction/executive	46.9 ± 7.6	53.3 ± 6.2	< 0.001^b^
Fine motor skills	43.6 ± 10.4	48.1 ± 5.4	0.012^b^
Attention/working memory	34.8 ± 6.8	47.4 ± 6.5	< 0.001^b^

The clinical data and neurocognitive test scores between the two groups were compared by a two-sample t-test or Mann–Whitney U test. Significance was set as p < 0.05. VL, viral load; HC, healthy control; ANI, asymptomatic neurocognitive impairment; ^a^ t-test; ^b^ Mann–Whitney U Test; and SD-Standard Deviation.

### Neurocognitive tests

This study conducted detailed neurocognitive tests in six cognitive domains using proven psychological test versions (26). These tests reflected differences in the corresponding cognitive field and were analyzed as follows: (1) verbal and language (animal naming test); (2) attention and working memory [Wechsler memory scale-III (WMS-III), and Paced Auditory Serial Addition Test (PASAT)]; (3) memory, including learning and recall [Hopkins verbal learning test-revised (HVLT-R) and brief visuospatial memory test-revised (BVMT-R)]; (4) speed of information processing [trail-making test Part A (TMT A)]; (5) fine motor skills (Grooved Pegboard Test); and 6) abstraction and executive function [Wisconsin Card Sorting Test 64-card version (WCST-64)]. The raw scores for all tests were converted to T scores and adjusted for age, gender, years of education, and the population size of the cities where the subjects grew up. If one cognitive domain contained multiple cognitive tests, the average T-score for that cognitive domain was calculated. Both the HIV-infected patients and healthy controls were provided with neurocognitive tests.

### MRI data acquisition

All imaging data were acquired on a 3.0T MR scanner (Tim-Trio, Siemens, Erlangen, Germany) using a 32-channel head coil. Tight foam pads were fixed to minimize movement during the scan. All subjects were instructed to lie quietly and relax, stay still, close their eyes but not fall asleep, and not think about anything in particular during the MRI scan. The rs-fMRI images were collected by using a gradient echo-planar imaging sequence. The acquisition parameters were as follows: repetition time (TR), 2,000 ms; echo time (TE), 30 ms; flip angle, 90°; resolution matrix, 64 × 64; field of view (FOV), 224 × 224 mm; section thickness, 3.5 mm; the number of sections, 35; and voxel size = 3.5 × 3.5 × 3.5 mm^3^. In total, 35 axial slices and 240-time points were obtained for each subject. In addition, a sagittal magnetization prepared gradient-echo (MPRAGE) sequence [repetition time (TR)/echo time (TE)/inversion time (TI) = 1,900/2.52/900 ms; acquisition matrix = 256 × 246; field of view = 250 × 250; flip angle = 9 degrees; voxel size = 1 mm × 0.977 mm × 0.977 mm] and axial T2-weighted fluid-attenuated inversion recovery (FLAIR) images (TR/TE/TI=8000/2370.9/97; and matrix, 320 × 224) were also acquired for the exclusion of overt brain diseases.

### Image preprocessing

Resting-state fMRI's were preprocessed using the data processing and analysis for brain imaging (DPABI V4.2) toolbox (27). The first 10 time points were removed to ensure the adaptation of the participants to the scanning environment and the equilibrium of the magnetic field. The remaining 230-time points were used for further slice-timing correction and realignment correction for head motion. Any subject with a translation of more than 2.0 mm or a rotation of more than 2.0° during the MRI scan was excluded from the study (28). Next, the resulting rs-fMRI data were spatially normalized to the Montreal Neurological Institute (MNI) space and resampled to a voxel size of 3 × 3 × 3 mm^3^. After that, to remove the effects of low-frequency drift and high-frequency physiological noises, all data were subjected to linear-trend discarding and temporal bandpass filtering (0.01 Hz < f < 0.08 Hz). In addition, the head motion parameters, white matter, and cerebrospinal fluid signals were regressed to reduce the possible adverse effects. The Friston 24-parameter model was performed to remove the head motion effects (29). The mean framewise displacement (FD) was added to the statistical model as a covariate in group analyses to reduce the residual effects of motion. Our study did not regress the global signal because some studies have shown that removing it may lead to an enhanced negative correlation or affect functional connectivity differences (30, 31). On the other hand, we also performed an analysis using fMRI data with global signal regression to validate the robustness of our findings, and these results have been added to the Supplementary materials.

### Computation of ReHo maps

ReHo maps of all subjects were calculated using the Kendall Coefficient of Concordance (KCC). Our study used the KCC to compute the ReHo of the time series within one voxel and its most adjacent 26 neighboring voxels (19). We obtained each subject's mean ReHo (mReHo) by dividing the ReHo value of each voxel in one ReHo map by the mean whole-brain ReHo value. Then, all mReHo maps were smoothed by a 4-mm full width at half the maximum Gaussian kernel. Significant differences between the two groups were identified on the mReHo maps using a two-sample *t*-test (*p* < 0.05). The results were analyzed by voxelwise false discovery rate (FDR) correction with a minimum cluster size of at least 10 contiguous voxels. Alternatively, based on the strictness of the group comparison used in this exploratory study, a threshold of *p* < 0.001 was applied in the conditional group comparison (uncorrected for multiple comparisons).

### ROI-based functional connectivity analysis

Based on the ReHo results above, we chose eight brain regions with significant group differences as the ROIs: the right lingual gyrus (LING. R), right superior occipital gyrus (SOG. R), left superior occipital gyrus (SOG. L), left middle occipital gyrus (MOG. L), right middle frontal gyrus (MFG. R), left middle frontal gyrus (MFG. L), left insula (INS. L), and vermis. To observe the changes in functional connectivity patterns, we constructed a pairwise connectivity matrix between these brain regions separately. This process uses DPABI software to calculate the functional correlation between the average time series of any two brain regions in different ROI. Through brain FC analysis, we obtained 8 × 7/2 ROI correlation coefficients for each subject. Then, we applied Fisher's Z transformation to the correlation coefficient matrix to improve the normality of the data (32). We used the two-sample *t*-test to calculate the intergroup differences in ROI correlation coefficients to compare the FC between the two groups. The threshold of all tests was set at *p* < 0.05, and FDR was used for multiple comparison corrections using the GRETNA software toolbox (33). The number of multiple comparison corrections in the ROI-based FC analysis was 28. In addition, considering that this study is exploratory and that the intergroup comparisons were strict, we also used the threshold of *p* < 0.001 for analyzing the data that did not pass FDR multiple comparisons.

### Statistical analysis

Two-sample *t*-tests and the Mann–Whitney U-test were used to analyze the group differences in demographics and clinical information. Multivariate linear regression was used to evaluate the neurocognitive test data (26). The significance threshold was set as *p* < 0.05. Statistical tests were performed in SPSS software (26.0).

The clusters showing significant group differences in ReHo and FC between the ANI and HC groups were visualized with the BrainNet Viewer (34) and DPABI software viewer. The mean ReHo values and FC correlation coefficient values of these abnormal clusters were extracted using DPABI software. Then, we applied Pearson's correlation analysis to examine the relationship between altered ReHo/FC and clinical variables (e.g., neurocognitive test scores and disease duration) in the ANI patients. Because this was an exploratory study, correlation analyses were not corrected by Bonferroni correction.

## Results

### Participants

The demographics, clinical characteristics, and neurocognitive test results of all patients and HCs are shown in Table 1. There were no significant differences in the demographics between the two groups. In our study, compared with healthy subjects, ANI groups showed significantly decreased T scores in all six cognitive domains.

### Differences in regional homogeneity

**Figure 1:**
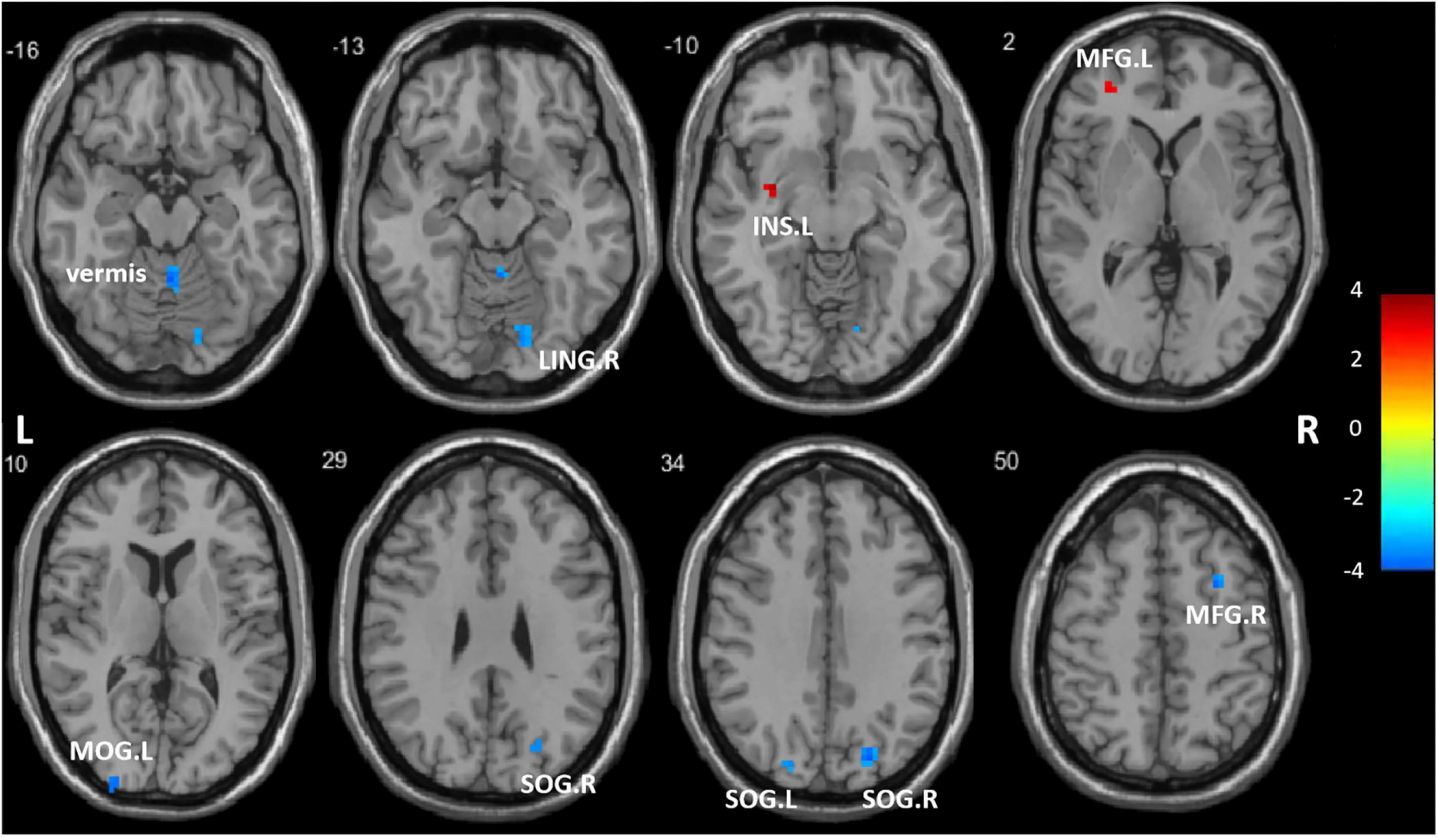
Regions showing significantly different regional homogeneity (ReHo) between the two groups. LING. R, right lingual gyrus; SOG. R, right superior occipital gyrus; SOG. L, left superior occipital gyrus; MOG. L, left middle occipital gyrus; MFG. R, right middle frontal gyrus; MFG. L, left middle frontal gyrus; INS. L, left insula; vermis. The color bar indicates a scale of T values.

A two-sample *t*-test showed significant group differences in ReHo between the ANI and HC groups in the following eight cortical and subcortical regions: Compared with HCs, the ANI patients had a significant ReHo decrease in the LING. R, SOG. R, SOG. L, MOG. L, MFG. R, and vermis, as well as ReHo enhancement in the MFG. L and INS. L (Figure 1, Table 2).

**Table 2:** Regional homogeneity differences between healthy controls and patients with ANI.

**Brain regions**	**Peak MNI, mm**			**T score**
	**X**	**Y**	**Z**	
Right lingual gyrus	12	−81	−12	−3.7
Vermis_4_5	0	−51	−15	−4.1
Left middle frontal gyrus	−33	48	6	3.5
Right middle frontal gyrus	27	9	51	−3.9
Right superior occipital gyrus	21	−78	33	−4.0
Left superior occipital gyrus	−21	−81	33	−3.5
Left middle occipital gyrus	−30	−99	9	−3.8
Left insula	−33	−3	−9	3.9

Coordinates (X, Y, Z) refer to the peak MNI coordinates of brain regions with peak intensity. A positive t value represents increased Reho, whereas a negative t value represents decreased Reho. The significance threshold was set at p < 0.001. MNI, Montreal Neurological Institute.

### Differences in ROI-based functional connectivity

**Figure 2:**
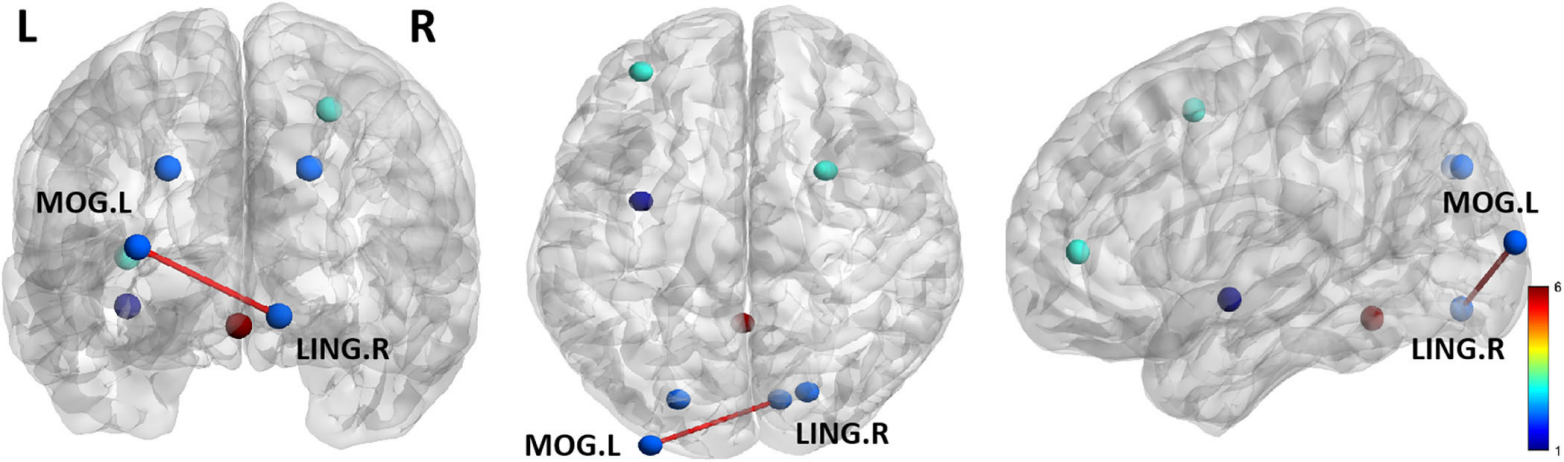
The two groups significantly differ in functional connectivity (FC) of ROI regions. Compared with HCs, the ANI patients showed significantly greater FC between the LING. R and MOG.L. Lines represent FC between each pair of defined ROI regions, with significant group differences between the two groups. The size of the lines represents the absolute value of the T values. LING. R, right lingual gyrus; MOG. L, left middle occipital gyrus.

A two-sample *t*-test showed significant group differences in FC between the two groups. As shown in Figure 2, compared with HCs, ANI patients showed significantly greater FC between the LING. R and MOG.L.

### Correlations of the imaging alterations with clinical variables and neurocognitive function

**Figure 3:**
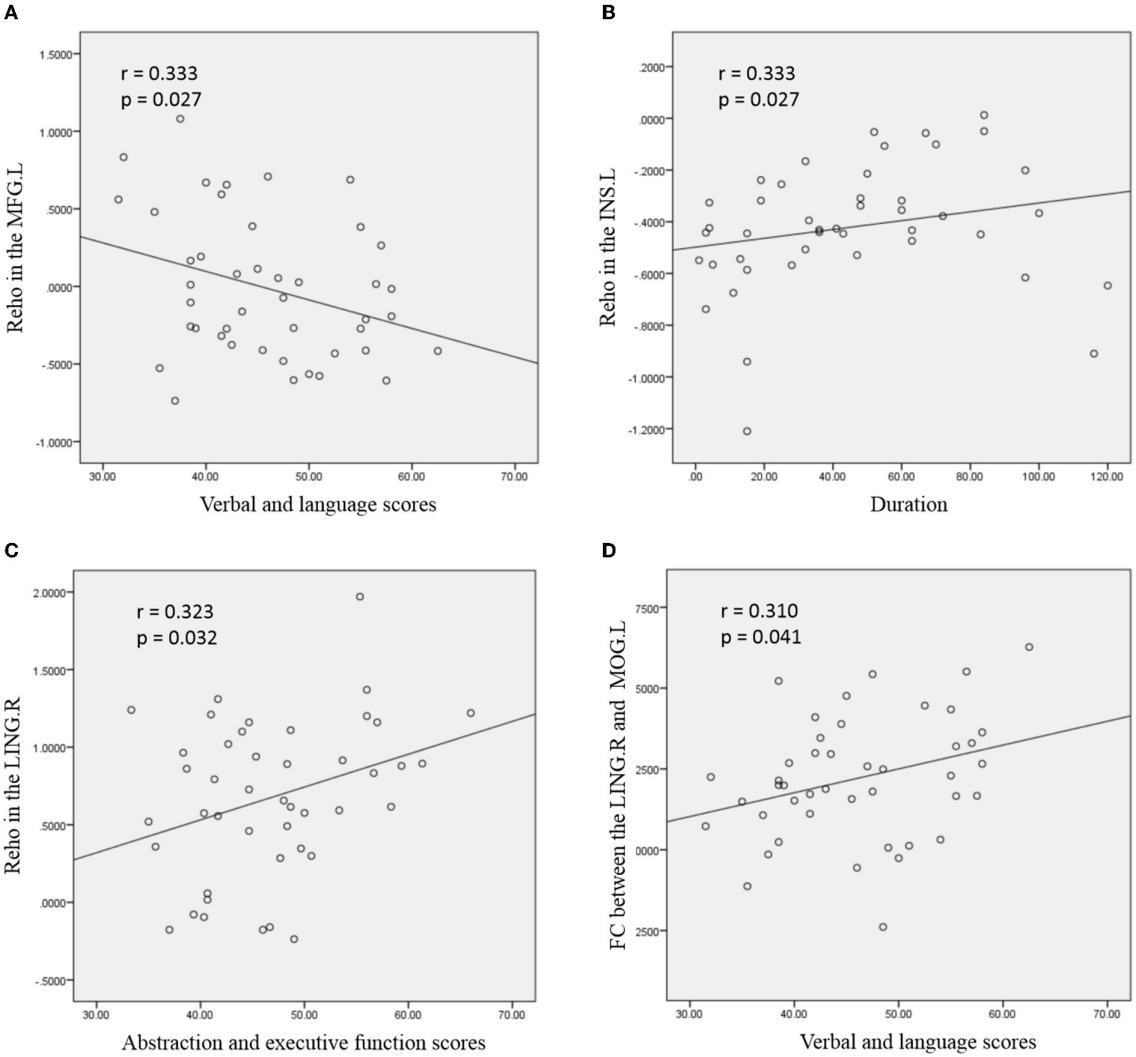
Correlation between abnormal regional homogeneity (ReHo)/functional connectivity (FC) and clinical variables in young HIV-infected patients with ANI. **(A)** depicts the mean ReHo in the left middle frontal gyrus (MFG. L), which was negatively correlated with the verbal and language scores; **(B)** shows the mean ReHo in the left insula (INS. L), which was positively correlated with the disease duration; **(C)** depicts positive correlations between mean ReHo in the right lingual gyrus (LING. R) and abstraction and executive function scores; **(D)** depicts the increased FC between the LING. R and left middle occipital gyrus (MOG. L), which was positively correlated with verbal and language performance. The threshold was set at *p* < 0.05 without multiple comparison corrections.

For ANI patients (Figure 3), the increased mean ReHo values in the MFG. L was negatively correlated with verbal and language scores (r = −0.319, *p* = 0.035). Increased mean ReHo values in the INS. L was positively correlated with the disease duration (r = 0.333, *p* = 0.027). The mean ReHo values in the LING. R were positively correlated with the abstraction and executive function scores in patients with ANI (r = 0.323, *p* = 0.032). Significantly increased z values of FC between the LING. R and MOG. L in ANI patients was also positively correlated with verbal and language performance (r = 0.310, *p* = 0.041). The abovementioned correlations were not corrected for multiple comparisons due to the relatively small patient group and the exploratory feature of this correlation analysis.

## Discussion

This study combined ReHo and ROI-based FC analysis to explore the patterns of local changes in brain regions and functional connectivity in young HIV-infected adults with ANI. Our study shows that young HIV-infected patients with ANI have different degrees of functional network abnormalities, which are related to some clinical features and neurocognitive abilities. These findings provide valuable information that furthers our understanding of the mechanism of functional network disorder in young HIV-infected patients.

In the present study, we found functional abnormalities in multiple regions of the visual network compared to HCs. This includes the LING. R, SOG. R, SOG. L, and MOG. L. The lingual gyrus is a brain structure involved in visual processing, especially related to letters (35). It is also considered to play a role in the logical analysis and encoding of visual memory (36). Decreased ReHo in the right lingual gyrus indicates alterations in logical analysis and visual processes in young HIV-infected adults. Some studies suggest that the right lingual gyrus is involved in abstraction and executive function (37), and the presence of reduced ReHo in this brain region suggests decreased visual network functioning. Abnormalities of the lingual gyrus have also been suggested to be correlated with impaired cognitive function (38). According to the correlation analysis, lower scores on WCST tests of young HIV-infected patients were correlated with reduced ReHo in the right lingual gyrus. We know that the WCST is a subset of the cognitive test battery used to evaluate abstraction and executive function (39). This result may suggest that young HIV-infected adults lack profound visual coordination activity.

The middle occipital gyrus and the superior occipital gyrus are located in the visual cortex. They are responsible for extracting visual information related to position and movement and emotional processing responses related to visual information (40, 41). They are also related to recognition memory. Our study showed that patients' ReHo values of the left middle occipital gyrus and the bilateral superior occipital gyrus decreased. Abnormalities in multiple regions indicate that the visual network of young HIV-infected patients has complex synergistic effects on task processing. In addition, the middle occipital gyrus is also considered an important node for integrating motor-related sensory information within the cortico-thalamo-cortical circuit (42). This means that as the disease progresses, damage to the visual network may affect other brain networks and functions through the thalamic circuit.

The cerebellar vermis is a major component of the spinocerebellar pathway, which receives messages from the spinal cord, controls muscle tone, and coordinates the maintenance of balance (43). This study found that the ReHo value of the cerebellar vermis in patients with ANI was lower than that in the HC group, suggesting that the neural activity of the cerebellar vermis in patients with ANI was decreased synergistically. Studies have shown that patients with HAND may develop balance disorders, such as abnormal gait, unsteadiness when standing, and side-to-side shaking as the condition worsens (44, 45). Our patients were in the stage of asymptomatic cognitive impairment and had no obvious clinical symptoms. However, we found local functional changes in the relevant brain regions. Thus, we speculated that reducing the ReHo value of the cerebellar vermis may reflect characteristic changes early in balance disorders and serve as a potential indicator for assessing disease-related progression. Of course, further experiments and follow-up studies are needed to verify this.

A notable finding in the present study was that the middle frontal gyrus is involved in regulating cognitive impairment during ANI. The middle frontal gyrus is associated with the integration of information, the allocation of cognitive resources, the maintenance of mental activity, and complex tasks. This brain region is also considered the control brain region (46). Our study showed that the ReHo value of the right middle frontal gyrus was decreased in ANI patients, while the ReHo value of the left middle frontal gyrus was increased. Unlike previous studies, these seemingly contradictory results may actually reflect compensatory regulation of abnormal brain regions (47). The middle frontal gyrus is also a cortical area controlling eye movement. It contains the path for eye movements, transmitting visual impulses and reciprocal connections. The abnormality of the visual network may affect the neurophysiological activity of the middle frontal gyrus through this visual control pathway. Correlative analysis showed the increased mean ReHo values in the MFG. L was negatively correlated with verbal and language scores. This result suggests the abnormality of MFG. L may affect the brain's ability to integrate language.

For young patients with ANI, the ReHo value in the left insular region was not only elevated but also positively correlated with the course of the disease. The insula is one of the main nodes of the salience network and plays an integrative role in cognitive-behavioral control. It also plays a role in various functions related to regulating emotional or physical homeostasis (48). Past studies have shown that the insula plays an important role in detecting salient external stimuli and regulating task-oriented cognitive control and is associated with a variety of neurological disorders, such as autism, schizophrenia, and depression (49–51). The increased ReHo in the insula may result from adaptive feedback to cognitive activity. The insula is the hub connecting the large-scale brain system. These hubs support all subjective feelings and participate in a series of cognitive, emotional, and sensory functions. Through correlation analysis, a positive relationship was found to exist between the ReHo value of the insula and the course of the disease. This suggests that abnormalities in the insula may reflect the disease progression of young HIV-infected patients with ANI.

Distant and local interconnections exist between different brain regions, forming brain networks (52, 53). To our knowledge, changes in regional synchronization and functional connectivity patterns in HIV-associated asymptomatic cognitive impairment have not been simultaneously investigated in previous rs-fMRI studies. Therefore, the present FC results may extend and confirm neuroimaging findings in young patients with ANI.

Compared with HCs, ANI patients showed increased FC between the right lingual gyrus and the left middle occipital gyrus, which was positively correlated with the verbal and language scores. The lingual and middle occipital gyrus belong to the visual network. This result suggests that cooperation within the visual network in patients with ANI is undergoing profound changes. The visual network is mainly responsible for controlling vision and visual information processing (36, 41). It comprises multiple adjacent functional areas that work synergistically to achieve complete visual processing (54). Damage to the visual network may promote the synergy of the functional overlapping brain regions, thereby achieving functional reorganization and compensation (55). In addition, correlation analysis suggested that this change may be driven by visual information and improved language performance in young ANI patients.

Our results suggest that visual networks play an important role in ANI. Both the results with and without the global signal regression showed the stability of the altered characteristics of the visual network in young ANI patients. Abnormalities in the visual network may affect the function of other brain regions closely related to visual information processing. Visual network performance is a promising key biomarker of asymptomatic neurocognitive impairment in young HIV-infected patients.

The current study has several limitations. First, stringent subject inclusion criteria resulted in our small sample size. This may affect the statistical power and interpretation of the final results. Second, the study was exploratory, as some results did not pass a strict multiple comparison correction and therefore required validation in larger studies. Third, this was a cross-sectional study, and we could not track changes in brain function during disease progression. A longitudinal study might reveal these alterations more powerfully and objectively.

## Conclusion

We demonstrated ReHo changes and abnormal FC in young HIV-infected patients with ANI through ReHo and ROI-based FC analysis. The results of this study suggest that the visual network represented by the lingual gyrus, superior occipital gyrus, and middle occipital gyrus might be the most vulnerable area of the brain in young HIV-infected patients with ANI. The middle frontal gyrus, cerebellar vermis, and insula also play an important role in asymptomatic neurocognitive impairment. Based on our analysis, we boldly speculate that visual network abnormalities may first drive the asymptomatic neurocognitive impairment in young HIV-infected patients. The ReHo and FC of these regions have multiple alterations that may be related to the course of the disease and neurocognitive function. These functional imaging findings will help us better understand the characteristics of brain network modifications in young HIV-infected patients with ANI.

## Data availability statement

The original contributions presented in the study are included in the article/Supplementary material, further inquiries can be directed to the corresponding authors.

## Ethics statement

The studies involving human participants were reviewed and approved by Beijing You'an Hospital Ethics Committee. The patients/participants provided their written informed consent to participate in this study. Written informed consent was obtained from the individual(s) for the publication of any potentially identifiable images or data included in this article.

## Author contributions

SH had full access to all of the data in the study and took responsibility for the integrity of the data and the accuracy of the data analysis and draft of the manuscript. Study concept and design: HL. Acquisition of data: SH, XA, JM, XY, and XW. Analysis and interpretation of data: SH and XA. Critical manuscript revision for important intellectual content: HL and LS. Study conception and design: JL and WW. Statistical analysis: SH and JL. All authors read and approved the final manuscript.

## Funding

This work was supported by the National Natural Science Foundation of China [Grant No. 61936013]; the Beijing Natural Science Foundation [7212051]. The funders and sponsors had no role in the study design, data collection, analysis, publication decision, or manuscript preparation.

## Conflict of interest

The authors declare that the research was conducted in the absence of any commercial or financial relationships that could be construed as a potential conflict of interest.

The handling editor JL declared a shared parent affiliation with the authors at the time of review.

## Publisher's note

All claims expressed in this article are solely those of the authors and do not necessarily represent those of their affiliated organizations, or those of the publisher, the editors and the reviewers. Any product that may be evaluated in this article, or claim that may be made by its manufacturer, is not guaranteed or endorsed by the publisher.
