# Supplementary material for: Altered regional homogeneity and functional connectivity of brain activity in young HIV-infected patients with asymptomatic neurocognitive impairment
Source: Front Neurol. 2022 Oct 10;13:982520. doi: 10.3389/fneur.2022.982520 (PMC9593212; doi:10.3389/fneur.2022.982520)
Supplement: Supplementary file 1 [file Table_1.DOCX]

Supplementary Material

The regional homogeneity results using fMRI data with globe signal regress are shown in the supplementary Table 1, which is highly consistent with the results without global signal regression. Compared with HC, the ANI patients had significantly ReHo decrease in the right lingual gyrus (LING.R), right superior occipital gyrus (SOG.R), left superior occipital gyrus (SOG.L), left middle occipital gyrus (MOG.L), triangular part of right inferior frontal gyrus (IFGtriang.R) and cerebellar vermis, as well as ReHo enhancement in the left middle frontal gyrus (MFG.L) and left dorsolateral superior frontal gyrus (SFGdor.L). The two results are consistent in the right lingual gyrus (LING.R), right superior occipital gyrus (SOG.R), left superior occipital gyrus (SOG.L), left middle occipital gyrus (MOG.L), left middle frontal gyrus (MFG.L) and vermis. Especially in the visual network area, the results of fMRI data without global signal regression are almost identical with the results of fMRI data with global signal regression. This analysis results validated the robustness of our discovery.

**Supplementary Table 1. Regional homogeneity differences between healthy controls and patients with ANI （using fMRI data with globe signal regress）**

| Brain regions | Peak MNI, mm | | | T score |
| --- | --- | --- | --- | --- |
|  | X | Y | Z |  |
| Right lingual gyrus | 12 | -81 | -12 | -3.5 |
| Vermis_4_5 | 0 | -48 | -12 | -3.9 |
| Left middle frontal gyrus | -33 | 45 | 3 | 3.6 |
| Right inferior frontal gyrus, triangular part | 60 | 33 | 9 | -3.9 |
| Right superior occipital gyrus | 21 | -78 | 33 | -4.4 |
| Left superior occipital gyrus | -21 | -81 | 36 | -3.5 |
| Left middle occipital gyrus | -27 | -99 | 9 | -3.6 |
| Left superior frontal gyrus, dorsolateral | -21 | 51 | 6 | 3.5 |

Coordinates (X, Y, Z) refer to the peak MNI coordinates of brain regions with peak intensity. Positive t value represents increased Reho, whereas negative t value represents decreased Reho. The significance threshold was set at p<0.001. Abbreviations: MNI, Montreal Neurological Institute.

Using the fMRI data with globe signal regress for analysis, the ANI patients showed increased FC between the LING.R and MOG.L compared to HC (supplementary Figure 1). This is consistent with the results without global signal regression.


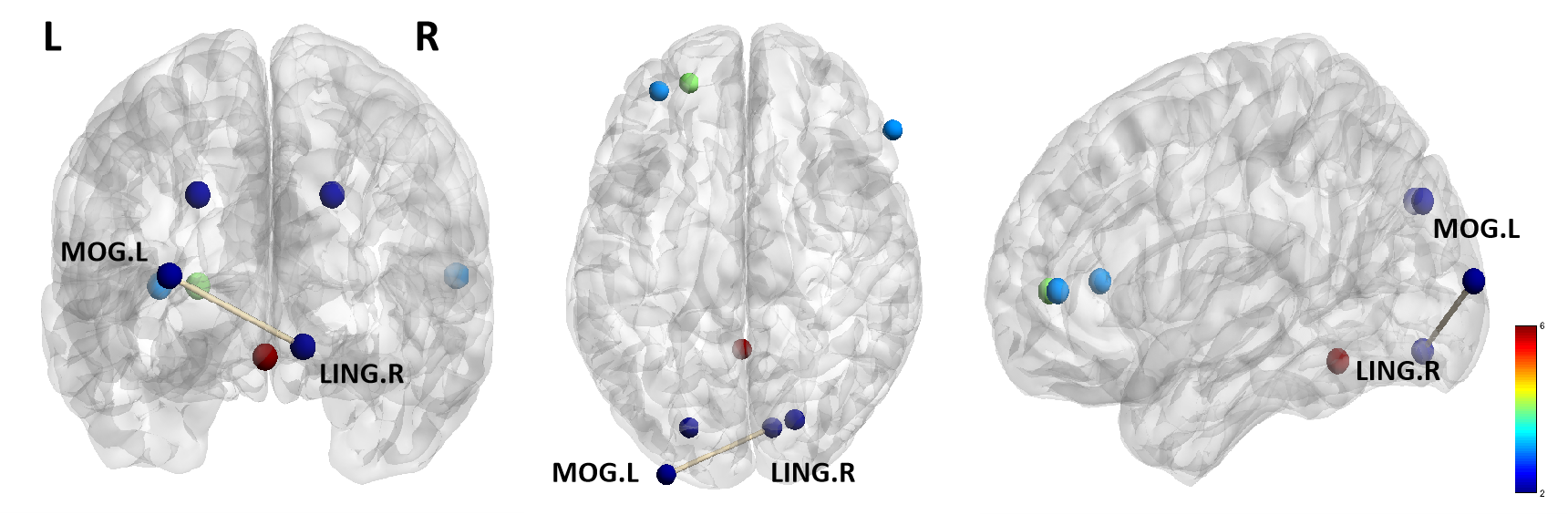


Supplementary Figure 1. Significant differences of functional connectivity (FC) of ROI regions between two groups. Compared with healthy controls, the ANI patients showed significantly greater FC between the LING.R and MOG.L. Lines represent FC between each pair of defined ROI regions with significant group differences between two groups. The size of lines represents the absolute value of T values. LING.R, right lingual gyrus; MOG.L, left middle occipital gyrus.
